# Depletion of Spleen Macrophages Delays AA Amyloid Development: A Study Performed in the Rapid Mouse Model of AA Amyloidosis

**DOI:** 10.1371/journal.pone.0079104

**Published:** 2013-11-13

**Authors:** Katarzyna Lundmark, Aida Vahdat Shariatpanahi, Gunilla T. Westermark

**Affiliations:** 1 Division of Experimental Pathology, Department of Clinical and Experimental Medicine, Faculty of Health Sciences, Linköping University; Pathology Clinic, County Council of Östergötland, Linköping, Sweden; 2 Department of Medical Cell Biology, Uppsala University, Uppsala, Sweden; Xavier Bichat Medical School, INSERM-CNRS - Université Paris Diderot, France

## Abstract

AA amyloidosis is a systemic disease that develops secondary to chronic inflammatory diseases Macrophages are often found in the vicinity of amyloid deposits and considered to play a role in both formation and degradation of amyloid fibrils. In spleen reside at least three types of macrophages, red pulp macrophages (RPM), marginal zone macrophages (MZM), metallophilic marginal zone macrophages (MMZM). MMZM and MZM are located in the marginal zone and express a unique collection of scavenger receptors that are involved in the uptake of blood-born particles. The murine AA amyloid model that resembles the human form of the disease has been used to study amyloid effects on different macrophage populations. Amyloid was induced by intravenous injection of amyloid enhancing factor and subcutaneous injections of silver nitrate and macrophages were identified with specific antibodies. We show that MZMs are highly sensitive to amyloid and decrease in number progressively with increasing amyloid load. Total area of MMZMs is unaffected by amyloid but cells are activated and migrate into the white pulp. In a group of mice spleen macrophages were depleted by an intravenous injection of clodronate filled liposomes. Subsequent injections of AEF and silver nitrate showed a sustained amyloid development. RPMs that constitute the majority of macrophages in spleen, appear insensitive to amyloid and do not participate in amyloid formation.

## Introduction

AA amyloidosis is a systemic disease that develops in patients with chronic infectious and inflammatory disorders, e.g. rheumatoid arthritis, familial Mediterranean fever, and tuberculosis [1], with renal failure as main clinical outcome. The main amyloid constituent in this form of amyloid disease is N-terminal fragments [2], [3] of the acute phase reactant, serum amyloid A (SAA) [4]. SAA is produced by hepatocytes in response to inflammatory cytokines (TNF-α, IL-1 and IL-6) and circulates in plasma associated with high-density lipoproteins (HDL) [5], [6]. Macrophages are often detected in close proximity to amyloid and considered significant for both the formation and degradation of aggregates. These processes appear to be independent of amyloid protein. Co-localization of SAA/AA to lysosomes of monocytoid cells in mice with reactive amyloidosis implicate a role for lysosomes in amyloid formation [7], [8], and *in vitro* studies have shown that SAA endocytosed by macrophages accumulates in intracellular vesicles and transform into amyloid [9]. Giant cells formed in AL amyloid frequently contain amyloid fibrils [10] and Kupffer cells phagocytose AA amyloid during amyloid clearance [7], [11], [12]. Injections of macrophage colony-stimulating factor in brain of transgenic mice that develop Alzheimer's disease led to increased number of microglia and decreased number of Aβ deposits [13].

Amyloid consists of protein fibrils whose formation occurs via a multistep process. The initial step is aggregation of monomers into nidus, which acts as starting point for fibril growth. The individual fibril grows when monomers are added to free ends and when long fibrils break this leads to increased number of free ends [14], [15], [16]. Nidus formation is probably the time determining step and the amyloid formation process can be accelerated from weeks to days by seeding with minute amounts of preformed fibrils, often referred to as amyloid enhancing factor (AEF). This works well for experimental induction of AA amyloidosis in mouse, hamster and mink [17], [18], [19]. In mouse, amyloid deposition starts in spleen followed by liver and thereafter a general distribution occurs. In spleen, early deposits can be detected in the marginal zones already within 48 hours after induction [7], [20], [21].

Spleen has a unique architecture and combines the function of blood filtration and innate and adaptive immunity [22]. The white pulp (WP) contains T-lymphocytes localized in periarterial lymphoid sheets and B-lymphocytes mature in germinal centre. WP is encircled by a marginal zone (MZ) formed by different types of specialized cells, among them marginal zone macrophages (MZMs) and metallophilic marginal zone macrophages (MMZMs) [23]. MZMs are localized to the outer part of marginal zone and characterized by expression of C-type lectin SIGNR1 and type I scavenger receptor MARCO [24]. MMZMs are localized to the inner part of the marginal zone in direct contact with the WP and express the adhesion molecule SIGLEC1 [25]. The two populations of macrophages are separated by a marginal sinus. Marginal zones are surrounded by red pulp (RP) containing red pulp macrophages (RPMs). RPMs are localized in the cords of the red pulp and they express F4/80 [26]. Neither MZMs nor MMZMs express F4/80.

Current investigations were undertaken to study the significance of different splenic macrophage populations in AA amyloidogenesis. The results show that MZMs are highly sensitive to amyloid and decrease progressively with increasing amyloid load. MMZMs remain unaffected by amyloid. Macrophage depletion with clodronate containing liposomes (CL) results in a significant reduction of amyloid formation. An incidental finding is that CL has AEF effect in inflamed mice.

## Results and Discussion

### Amyloid Induction

**Figure 1:**
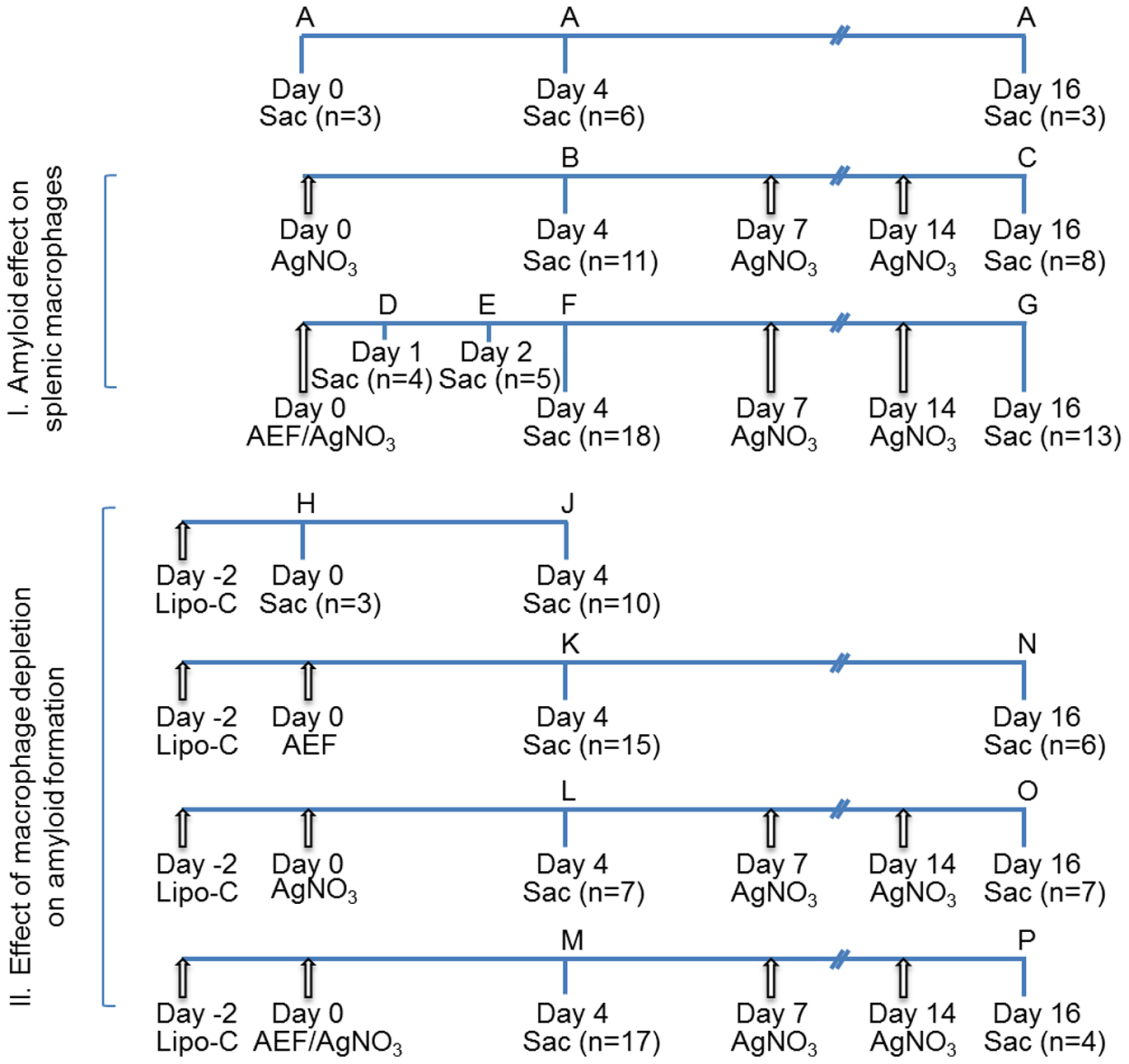
The cartoon outlines animal groups, treatment and time schedules used for the two studies. In section I, is ¨Effect of amyloid on splenic macrophages¨ described, and in II, is ¨Effect of macrophage depletion on amyloid formation¨ described. Capital letter indicates experimental group, arrows indicate time points for injections, Sac indicate time points when mice were sacrificed, and n specifies number of mice.

To our knowledge, this is the first study of spleen amyloid formation that pinpoints the significance of different spleen macrophages. Effects of AA amyloid on spleen macrophages were studied using the rapid mouse model as outlined in figure 1.I. Spleen, liver and serum samples were collected and stored at −80°C, until used. Frozen sections (10 µm) from spleen were stained for amyloid with alkaline Congo red. Amyloid load was graded according to following scheme adapted from Lundmark et al. [27]: 0, no amyloid; 1+, minimal amyloid aggregates in single marginal zones; 2+, small amyloid deposits occupying part of the marginal zone in several lymphoid follicles; 3+, moderate amyloid deposits around most or all follicles; 4+, extensive amyloid deposits around most or all follicles with continuous deposits in the red pulp.

Mice in control groups were untreated (group A, n = 12) or given 1 or 3 AgNO_3_ injections and sacrificed after 4 and 16 days, respectively (groups B, n = 11 and C, n = 8). Amyloid was induced in 40 female NMRI mice with AEF and AgNO_3_ injections and animals were killed at different time points. Over the years we have experienced variations in amyloid induction and ascribe this to improved animal health and care. Therefore, we started to determine the initial time point for amyloid formation and mice were killed 1 day (group D, n = 4) and 2 days (group E, n = 5), after induction. No amyloid was detected in spleen sections from the 4 mice in group D while amyloid was detected in 1 out of 5 mice in group E (Table 1). In mice killed 4 days after induction, amyloid was detected in marginal zones in 17 out of 18 mice (group F). The amyloid load varied and was graded 1+ to 4+ (Table 1). All mice killed 16 days after induction (group G) had amyloid and extensive deposits (4+) were detected in 11 out of 13 mice (Table 1). Amyloid was also detected in liver from 11 out of 17 mice with splenic amyloid from group F and in all mice from group G.

**Table 1:** Grading of spleen amyloid and quantification of RPMs and MMZMs during amyloid development.

Group	Treatment	Sacrificedday	No. of mice/no.of mice withamyloid	Amyloidgrade	% increase ofRPMs±SDcompared tountreated mice	% change of MMZMs±SDcompared tountreated mice
B	AgNO_3_	4	11	0	35±10***	↓ 1±33
C	AgNO_3_	16	8	0	25±6**	↑ 15±11
D	AEF+AgNO_3_	1	4	0	32±6**	↑ 7±30
E	AEF+AgNO_3_	2	5/1	2+	33±7**	↓ 3±18
F	AEF+AgNO_3_	4	18/17	1+−4+	22±10**	↑ 10±19
G	AEF+AgNO_3_	16	13/13	3+−4+	26±19*	↑ 2±15

See figure 1 for definition of groups, *p<0.05; **p<0.01; ***p<0.001. Presence of spleen amyloid was analysed after Congo red staining and RPMs and MMZMs were detected with antibodies specific for F4/80 and MOMA-1, respectively.

↓ indicate decrease and ↑ indicate increase of MMZMs, respectively.

We conclude that under the given circumstances amyloidogenesis starts almost 2 days before amyloid can be demonstrated in cross polarized light after Congo red staining. Since only animals with splenic deposits exhibited liver amyloid (group F and G), spleen marginal zone seems to be the initial amyloid deposition site. Our results are in accordance with results from earlier studies using this rapid mouse model for AA-amyloidosis [17].

### Amyloid Deposition is Accompanied by Changes of Macrophages

#### RPMs

**Figure 2:**
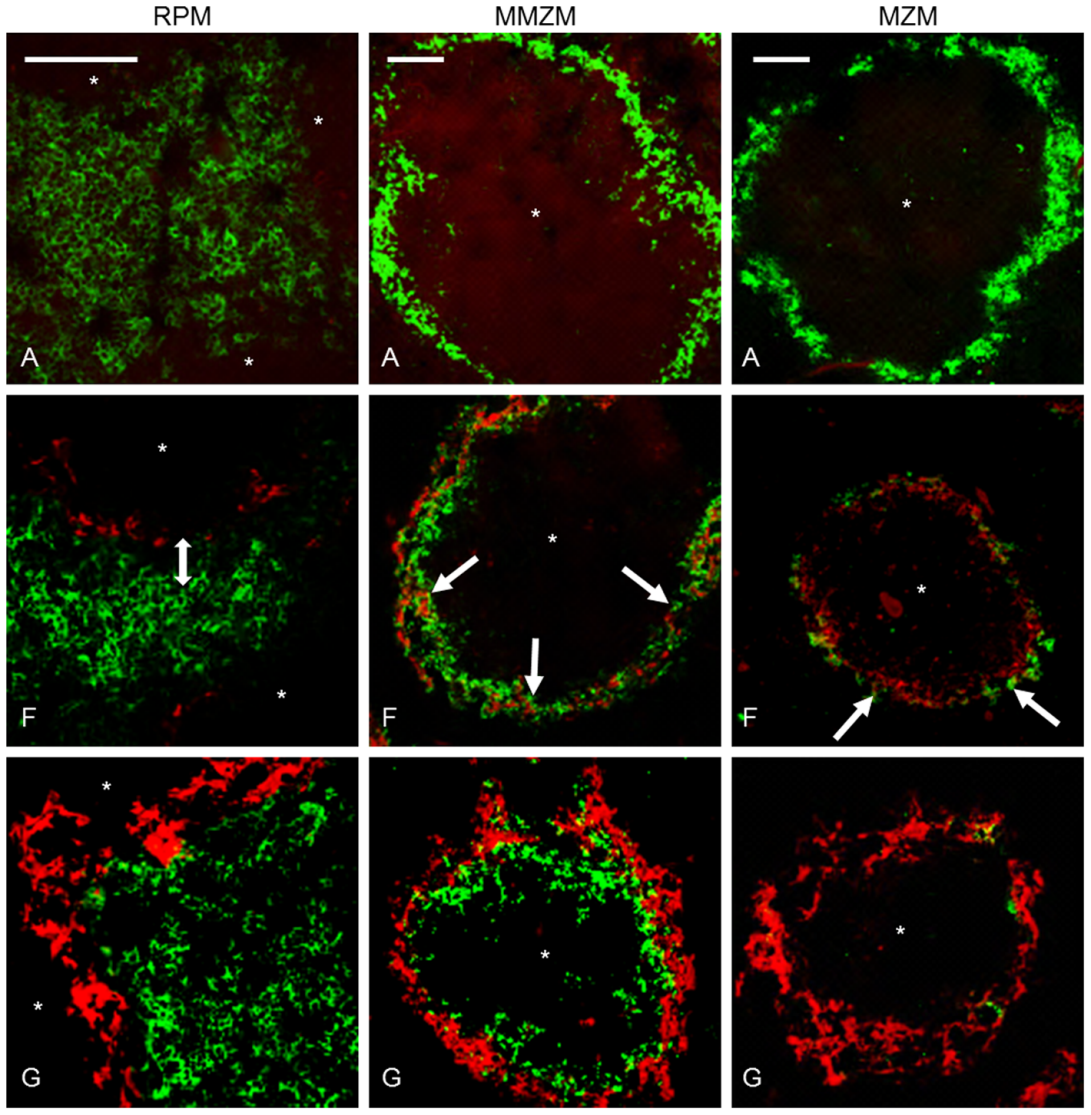
Amyloid deposition was accompanied by changes of splenic macrophages. Group A untreated control, group F received AEF and AgNO_3_ day 0 and was sacrificed day 4, group G received AEF and AgNO_3_ day 0, additional injections of AgNO_3_ on day 7 and 14, and was sacrificed day 16. In the left panel, a double arrow indicates a distance between RPMs and amyloid in group F, which does not persist in group G. The middle panel depicts MMZMs intermingling with amyloid in groups F (white arrow) and G. In the right panel, arrow indicates an onset loss of MZMs in group F, and complete loss of MZMs in marginal zones with extensive amyloid deposits in group G. Capital letter refers to experimental group, amyloid in red, macrophages in green,* indicate white pulp region, and bar = 100 µm.

**Figure 3:**
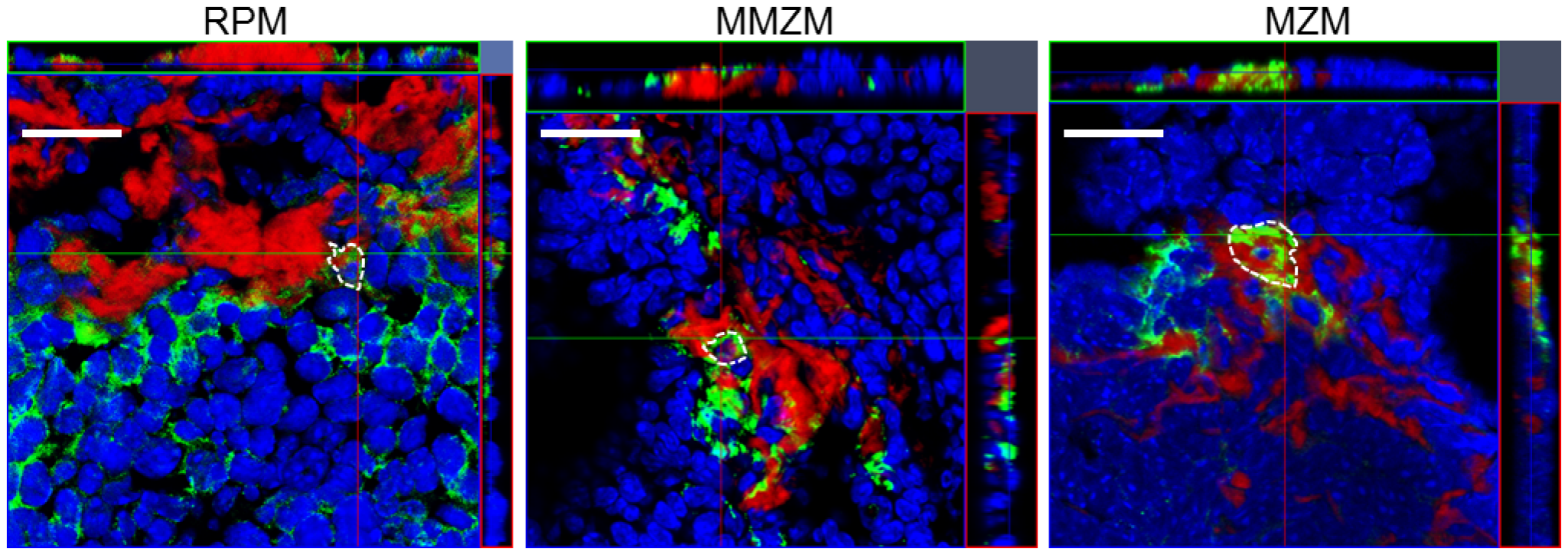
Intracellular amyloid was detected in RPMs, MMZMs and MZMs. Frozen sections from group G treated with AEF and AgNO_3_ on day 0 and additional injections of AgNO_3_ on day 7 and 14, sacrificed on day 16 were immunolabelled with F4/80 to detect RPMs, with MOMA-1 to detect MMZMs or with ERTR-9 to detect MZMs. Amyloid was detected by incubation in Congo red solution B. Amyloid is showed in red, macrophages in green and white dotted line encircles amyloid containing cells. Bar = 20 µm.

We studied amyloid effects on RPMs, MMZMs and MZMs using specific antibodies against F4/80, MOMA1 or ER-TR9, respectively (see material and methods), combined with Congo red. Spleens recovered from untreated mice (group A) were used as a reference for normal content and distribution of macrophages. F4/80 labelling of RPMs revealed an even distribution of these cells in the red pulp in untreated mice (Figure 2 RPM-A). F4/80 labelled areas were measured in sections from at least 3 randomly selected mice from groups A–G The area of RPMs increased 22–35% in groups B–G (Table 1). Sections from animals in group A, B and D were stained with proliferation marker Ki67, and Ki67 labelling index (^Ki67^LI) was calculated as percentage of Ki67 positive cells of total number of RPM/2000 µm^2^. ^Ki67^LI was determined to 76±19, 96±3 and 94±8 in groups A, B and D, respectively. This means that the observed increase depend on proliferation and is most likely an acute phase response caused by AgNO_3_ and independent of amyloid formation. Early during amyloid deposition a distance between the amyloid in the marginal zone and RPMs was observed (Figure 2 RPM-F). When amyloid mass advanced, a direct contact with RPMs was seen (Figure 2 RPM-G). Gathering of RPMs at the marginal zones with extensive amyloid deposits might result from an expansion of the marginal zone, or more interestingly, migration of RPMs toward the amyloid. RPMs role in amyloidosis is complex and F4/80 positive macrophages have been shown to process SAA into AA-amyloid *in vitro* [28] as well as degrade amyloid [7], [11], [12], [29]. However, the observed distance between RPMs and the amyloid in the marginal zone early during amyloid deposition argues against RPMs as the target cells for amyloidgenesis in this AA-amyloid model. Also, minimal intracellular amyloid deposits could be detected in few RPMs late during amyloid deposition (group G), and in this context, they were more likely a result of phagocytosis and indicative of degradation (Figure 3, RPM).

#### MMZMs and MZMs

MMZMs and MZMs revealed strong cytoplasmic expression of their specific markers MOMA-1 and ER-TR9, respectively. In spleen from untreated mice (group A) the marginal zone appeared as a continuous ring-like structure with MMZMs in the inner rim adjacent to the white pulp, and MZMs in the outer rim adjacent to the red pulp (Figure 2, MMZM-A and MZM-A).

The total area of labelled MMZMs was not affected by AgNO_3_ or amyloid deposits (Table 1). In marginal zones with amyloid MMZMs appeared partially intermingled with the deposits (Figure 2 MMZM-F and MMZM-G) and minute amounts of amyloid could be detected in few cells (Figure 3, MMZM).

Interestingly, we observed migration of MMZMs from the marginal zone into the white pulp in spleen recovered 16 days after amyloid induction (Figure 2 MMZM-G). AA-amyloidosis in mice is associated with the appearance of autoantibodies against protein AA. It is possible that migrating MMZMs are initiating formation of such antibodies [12]. This migration was not seen in marginal zones absent of amyloid, indicating that it is a response to amyloid. A similar phenomenon was described to occur after injection of *Listeria monocytogenes* and this was suggested to up-regulate chemokine receptor CXCR5 responsible for recruitment of B cells the white pulp [30].

MZM areas increased in response to inflammatory stimulation with AgNO_3_ by 7–9% in groups B and C (Table 2). Administration of AgNO_3_ and AEF in groups D, E and F lead to a further increase of MZM areas up to 66% (group D, Table 2). Quantification of cell nuclei in MZM areas showed that there was also an increase in number of cells by 4, 35, 18 and 15% in groups B, D, E and F, respectively. This suggests marked cell proliferation, especially in response to AEF. The faith of MZMs changed dramatically when amyloid started to form and in marginal zones with amyloid an increasing amyloid load led to a progressive reduction of MZMs (groups E–G. In group G, 5 out of 11 mice with amyloid score 4+ were totally depleted of MZMs (Table 2, Figure 2 MZM-G) while remaining 6 animals with score 4+ contained only few MZMs, all with abundant intracellular amyloid (Figure 3 MZM).

**Table 2:** Grading of spleen amyloid and quantification of MZMs during amyloid development.

Group	Treatment	Sacrificeday	No. ofmice	No. of mice withamyloid/amyloidgrade	No. micewith MZM	% decrease of MZM±SDin MZ with amyloid	% increase of MZM±SDin MZ without amyloid
B	AgNO_3_	4	11	0/0	11	–	9±25
C	AgNO_3_	16	8	0/0	8	–	7±13
D	AEF+AgNO_3_	1	4	0/0	4	–	66±34*
E	AEF+AgNO_3_	2	5	1/2+	1	24±24*	26±54
					4	–	16±10
F	AEF+AgNO_3_	4	18	1/0	1	–	18±35
				8/1+	8	ND	35±23**
				3/2+	3	53±9***	49±24*
				5/3+	5	61±6**	#
				1/4+	0	100***	#
G	AEF+AgNO_3_	16	13	2/3+	2	80±8***	#
				6/4+	6	92±10***	#
				5/4+	0	100***	#

See figure 1 for definition of groups. *p<0.05; ** p≤0.01; *** p≤0.001.

Presence of antibodies specific for ER-TR9.

ND = not determined, MZ = marginal zone and # = no marginal zones without amyloid.

**Figure 4:**
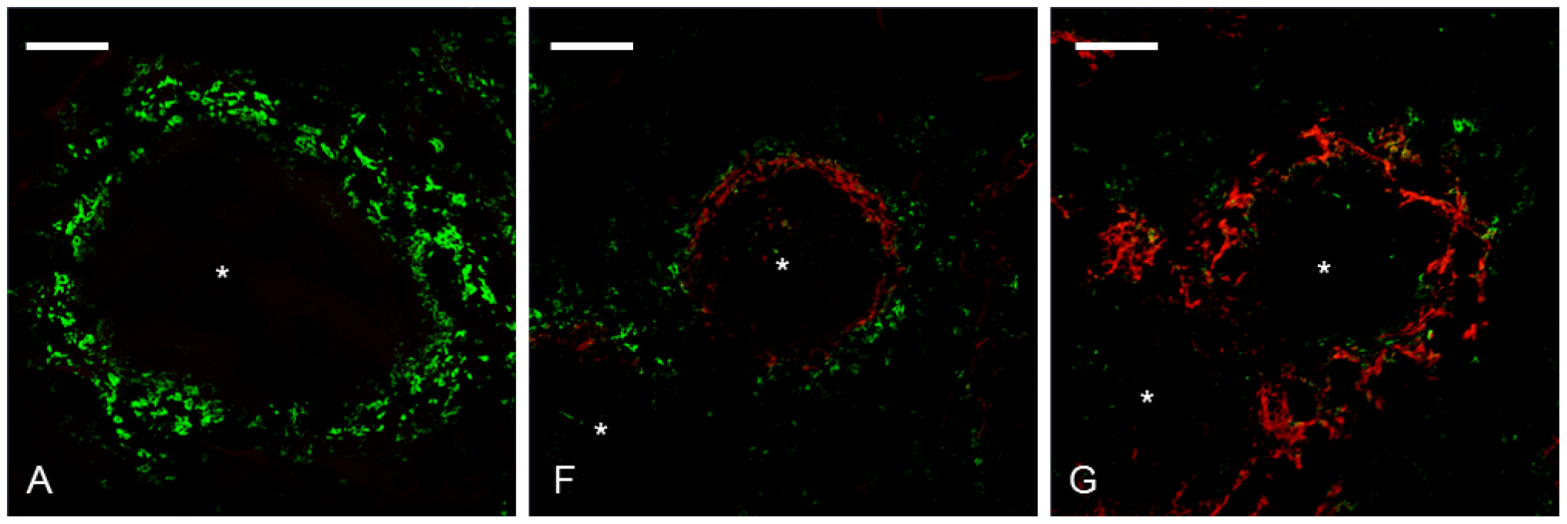
Amyloid deposition was accompanied by depletion of MZMs. Spleen sections from group A untreated control, group F received AEF and AgNO_3_ day 0 and was sacrificed day 4, group G received AEF and AgNO_3_ day 0, additional injections of AgNO_3_ on day 7 and 14, and was sacrificed day 16 were subjected to immunolabelling with antiMARCO antibodies. The left panel shows MZM content in spleen from group A. The middle panel shows reduction of MZMs (group F) and the right panel shows an almost complete loss of MZMs in marginal zones with extensive amyloid deposits (group G). Capital letter refers to experimental group, amyloid in red, MZMs in green, * indicate white pulp region, and bar = 100 µm.

Since amyloid deposition resulted in an almost complete eradication of MZMs, an additional immunolabelling was performed with antiMARCO antibodies on sections from groups A, F and G. The immune pattern obtained for MARCO was identical to that showed for ER-TR9 and confirms that amyloid deposition lead to a loss of MZMs (Figure 4).

MMZMs and MZMs have large phagocytic capacity but the latter are more exposed to blood-born particles because of their position in the marginal zone [31], [32]. It is therefore possible that the observed initial increase of MZMs in part reflects a response to injected amyloid fibrils while amyloid propagation leads to cell death. Earlier in vitro studies have shown that initial amyloid formation take place in macrophages and in association with lysosomes [33]. This finding may be in accordance with the present study in which both MMZMs and MZMs contained intracellular amyloid. However, if depletion of MZMs depends on intra- or extracellular amyloid is unknown, but the presence of intracellular amyloid in remaining MZMs supports an intracellular route of cytotoxicity.

### Depletion of Spleen Macrophages with Clodronate

Multilayered clodronate containing liposomes (CL) were prepared according to the method given by van Rooijen [34]. Clodronate is a bisphosphonate drug that activates apoptosis [35]. When liposome enclosed clodronate is injected intravenously, it will be phagocytosed by macrophages and this strategy is frequently used for specific depletion of spleen and liver macrophages. One intravenous injection of 0.2 ml CL is sufficient to eliminate all spleen macrophages within 24 hours [36]. CL with a diameter of 0.8–10 µm was injected intravenously in a group of 69 mice (day −2) (Figure 1.II.) Mice were divided into 8 subgroups (H, J, K, L, M, N, O and P). Mice in groups H (n = 3) and J (n = 10) were not exposed to any additional treatment and were sacrificed day 0 and 4, respectively. Mice in groups K (n = 15) and N (n = 6) received an intravenous injection of 0.1 ml AEF day 0, and were sacrificed day 4 and 16, respectively. Mice in groups L (n = 7) and O (n = 7) received a subcutaneous injection of 0.2 ml 1% AgNO_3_ day 0_,_ and mice in group O received additional injections of 0.1 ml 1% AgNO_3_ day 7 and 14, and animals were sacrificed day 4 and 16, respectively. Mice in group M (n = 17) and P (n = 4) received an intravenous injection of 0.1 ml AEF concomitant with a subcutaneous injection of 0.2 ml 1% AgNO_3_ and mice in group P received additional injections of 0.1 ml 1% AgNO_3_ day 7 and 14, and animals were sacrificed day 4 and 16, respectively.

The result of macrophage depletion was determined after immunolabelling (Table 3 and 4).

**Table 3:** Grading of spleen amyloid and quantification of RPMs after macrophage depletion.

Group	Treatment	Sacrificed day	No. of mice/no. of mice with amyloid	Amyloid grade	% of RPMs±SD
A	Untreated	0, 4, 16	12/0	0	100
H	CL	0	3/0	0	0±0***
J	CL	4	10/0	0	40±19***
K	CL+AEF	4	15/0	0	54±18***
L	CL+AgNO_3_	4	5/0	0	36±10***
M	CL+AEF+AgNO_3_	4	17/9	1+	52±15***
N	CL+AEF	16	6/1	2+	99±6
O	CL+AgNO_3_	16	7/7	2+	118±1*
P	CL+AEF+AgNO_3_	16	4/4	2+−4+	107±3

See figure 1 for definition of groups, *p<0.05; *** p≤0.001.

Presence of spleen amyloid was analysed after Congo red staining and RPMs were detected with antibodies specific for F4/80, CL = Clodronate containing liposomes.

**Table 4:** Grading of spleen amyloid and quantification of MMZMs after macrophage depletion.

Group	Treatment	Sacrificed day	No. of mice/no. of mice with amyloid	Amyloid grade	No. of micewith MMZMs	% of MMZM±SD
A	Untreated	0, 4, 16	12/0	0	12	100
H	CL	0	3/0	0	2	2±2**
J	CL	4	10/0	0	2	2±2**
K	CL+AEF	4	15/0	0	9	2±2**
L	CL+AgNO_3_	4	5/0	0	2	1±2**
M	CL+AEF+AgNO_3_	4	17/9	1+	7	2±5**
N	CL+AEF	16	6/1	2+	6	82±16
O	CL+AgNO_3_	16	7/7	2+	7	72±1
P	CL+AEF+AgNO_3_	16	4/4	2+−4+	4	79±17

See figure 1 for definition of groups, ** p≤0.01. Presence of spleen amyloid was analysed after Congo red staining and MMZMs were detected with antibodies specific for MOMA1, CL = Clodronate containing liposomes.

#### RPMs

**Figure 5:**
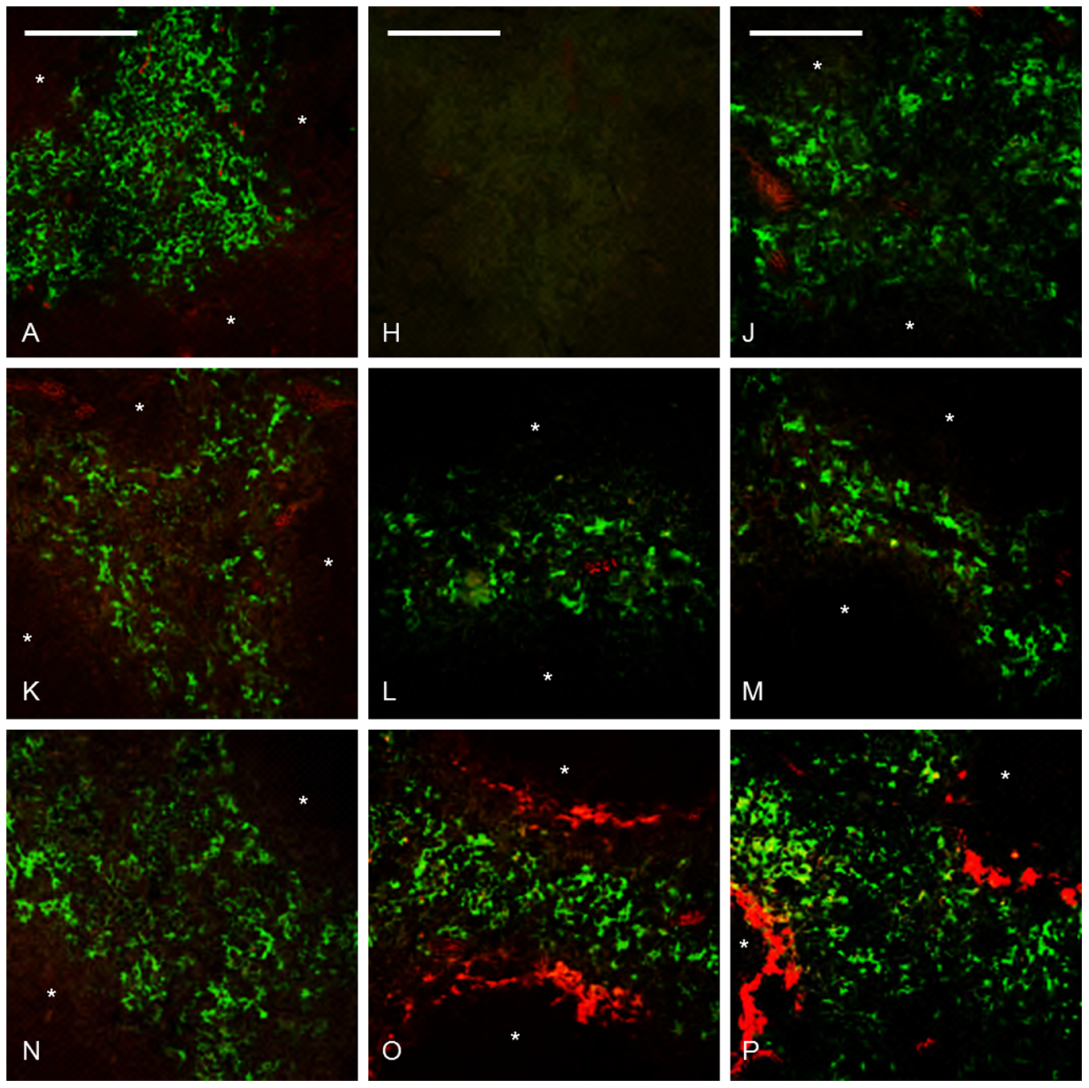
RPMs were partially recovered 4(J–M and their presence did not affect amyloid development. Group A untreated control, groups H and J received CL day -2, and were sacrificed day 0 and day 4, respectively. Groups K and N received CL day -2 and AEF day 0, and were sacrificed day 4 and day 16, respectively. Groups L and O received CL day -2 and AgNO_3_ day 0, group O received additional injections of AgNO_3_ day 7 and 14, and were sacrificed day 4 and day 16, respectively. Groups M and P received CL day -2 and AgNO_3_ and AEF day 0, mice from group P received additional injections of AgNO_3_ on day 7 and 14, and were sacrificed day 4 and16, respectively. Capital letter refers to experimental group, amyloid in red, RPMs in green, and bar = 100 µm.

An intravenous injection of 0.2 ml CL led to total depletion of RPMs in mice killed 2 days after injection (group H, Table 3, Figure 5H). RPMs reappeared already 6 days after CL injection, occupying 36 to 54% of the original area (groups J, K, L, M, Table 3, Figure 5J–M). In mice sacrificed 18 days after CL treatment RPMs were reestablished in groups N, O and P (Table 3, Figure 5N–P. The reappearance of RPMs was independent of additional injections of AEF, AgNO_3_ or AEF and AgNO_3_ after CL treatment.

#### MMZMs and MZMs

Two mice sacrificed 6 days after CL injection (Group L) were excluded from the study, since they exhibited normal amounts of MMZMs but no MZMs. This shows that MMZMs are partially protected by their localization to the inner region of the marginal zones and can remain if exposed to an insufficient dose of CL.

**Figure 6:**
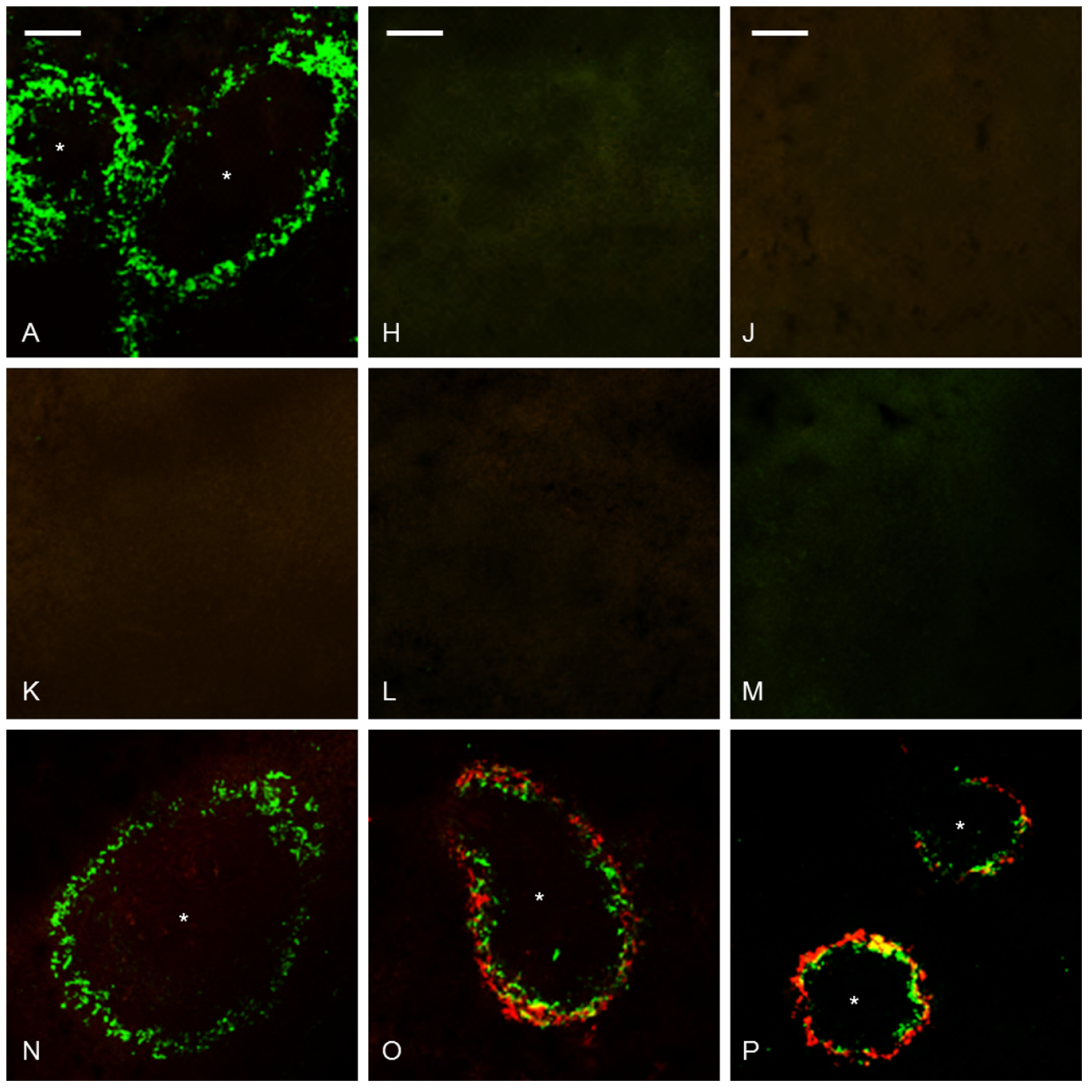
Depletion of MMZMs resulted in delayed amyloid formation, and MMZMs were re-established day 16, a time point when amyloid was demonstrated (group P). Amyloid appeared also in all mice from group O which after macrophage depletion received AgNO3 day 0, 7 and 14 and sacrificed day 16. This treatment does not normally lead to amyloid depositions. Intracellular amyloid co-localize with MMZMs (yellow) is seen in marginal zones of mice from groups O and P. Group A untreated control, groups H and J received CL day -2, and were sacrificed day 0 and day 4, respectively. Groups K and N received CL day -2 and AEF day 0, and were sacrificed day 4 and day 16, respectively. Groups L and O received CL day -2 and AgNO_3_ day 0, group O received additional injections of AgNO_3_ day 7 and 14, and were sacrificed day 4 and day 16, respectively. Groups M and P received CL day -2 and AgNO_3_ and AEF day 0, mice from group P received additional injections of AgNO_3_ day 7 and 14, and were sacrificed day 4 and day 16, respectively. Capital letter refers to experimental group, amyloid in red, MMZMs in green, * indicate white pulp region and bar = 100 µm.

Single MMZMs remained in few marginal zones in 2 out of 3 mice examined 2 days after CL injection (group H, Table 4, Figure 6H). In the 47 mice sacrificed 6 days after CL injection were MMZMs eradicated in 27, and did not exceed 2.0% of the original area in the remaining 20 mice (groups J, K, L, M, Table 4, Figure 6 J–M. In the 17 mice sacrificed 18 days after CL treatment MMZMs reappeared and occupied almost 72–82% of the original area (groups N, O and P, Table 4, Figure 6N–P.

**Figure 7:**
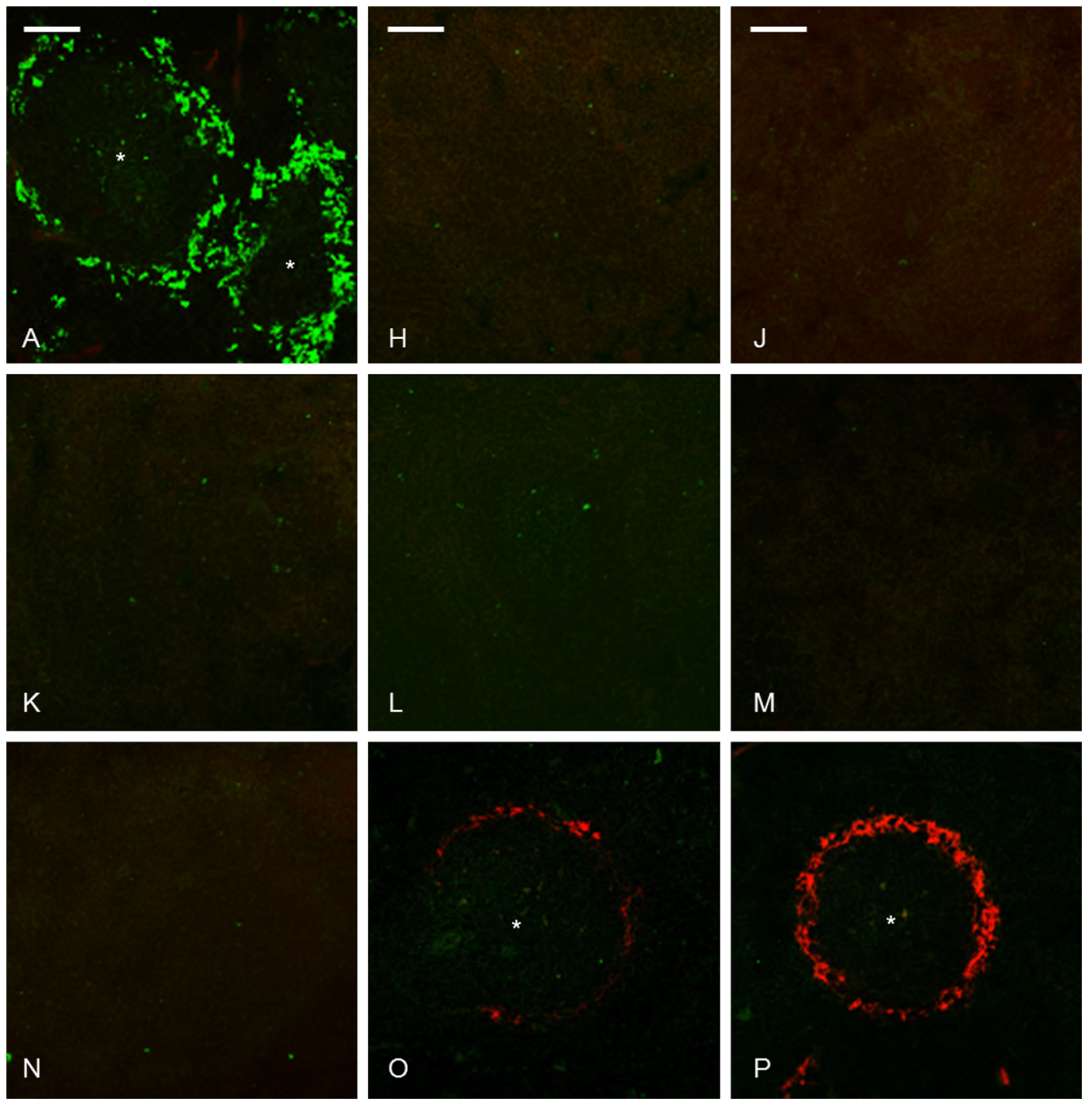
Abolition of MZMs did not influence amyloid development and MZMs were still absent day 16 when amyloid was demonstrated in groups O and P. Group A untreated control, groups H and J received CL day -2, and were sacrificed day 0 and day 4, respectively. Groups K and N received CL day -2 and AEF day 0, and were sacrificed day 4 and day 16, respectively. Groups L and O received CL day -2 and AgNO_3_ day 0, group O received additional injections of AgNO_3_ day 7 and 14, and were sacrificed day 4 and day 16, respectively. Groups M and P received CL day -2 and AgNO_3_ and AEF day 0, mice from group P received additional injections of AgNO_3_ day 7 and 14, and were sacrificed day 4 and day 16, respectively. Capital letter refers to experimental group, amyloid in red, MZMs in green, * indicate white pulp region and bar = 100 µm.

MZMs were almost completely depleted from the outer region of the marginal zones after CL injection. Single MZMs were identified in few marginal zones in 8 out of 67 mice, but this area did not reach 0.1% of the area determined in untreated control mice (group H–P Figure 7H–P.

### Depletion of Macrophages Decreases Amyloid Incidence and Load

Injection of AEF+AgNO3 in mice that were sacrificed 4 days later lead to amyloid development in 9 out of 17 mice depleted of macrophages compared to 17 out of 18 mice with macrophages (group M, Table 3 and group F, Table 1) (P = 0.00006). Amyloid did not develop in macrophage-depleted mice from other groups (groups J, K and L, Table 3) sacrificed at that time point. All mice depleted of macrophages and sacrificed 16 days after amyloid induction developed amyloid, scored 2–4+ (group P, Table 3, Figure 5P, 6P and 7P). This is less compared to mice in group G, all with macrophages and where almost all mice developed 4+ deposits. Liver amyloid was detected in 6 out of 9 mice in group M and in all mice from groups O and P. Mice without splenic amyloid did not develop liver deposits.

Our results show that MZMs are not required for amyloid formation, but their depletion lead to delayed amyloid occurrence and reduced incidence. These macrophages express a large number of surface molecules such as scavenger receptor A (SR-A), macrophage receptor collagenous (MARCO) and SIGN-related (SIGNR1) with capacity of recognition and phagocytosis of blood-born particulate antigens [37]. It is therefore possible that the above described activation of MZMs by AEF is a result from a specific interaction with a receptor from one of these classes. AEF constitutes of short amyloid fibrils [38] and if phagocytosed but not degraded these can act as nidus for intracellular amyloid growth. A subsequent emergence of amyloid mass may lead to MZM cell death. After macrophage depletion, delayed amyloid formation still occurred in the marginal zones. It is therefore possible that AEF fibrils became trapped by remaining MZM and MMZM debris or by the reticular network and could still function as seeds for amyloid growth, but now with a much lower efficiency.

In the 2 mice injected with insufficient dose of CL MMZMs remained while RPMs and MZMs were eradicated. This confirms that blood flow and localization in sinus give MMZMs an initial protection from blood-borne particulate. Also, in 20 mice out of 47 sacrificed 6 days after CL injection 1–2% of MMZMs remained in the marginal zones and in group M early amyloid deposits were only detected in mice with MMZMs (Table 4). The amyloid mass increased parallel with MMZMs reappearance and in groups O and P was intracellular amyloid frequently demonstrated (Figure 6O and P), unlike group (G) in which only minimal deposits could be detected in few cells. This indicates a role for MMZMs in the amyloid formation in the absence of MZMs. However, this mechanism must differ from the mechanism used by MZMs since MMZMs survive amyloid formation. One can speculate that AEF binds to receptors on the surface of macrophages and that AEF then binds SAA-protein, leading to fibril growth mainly extracellularly as suggested for AL amyloidosis [39] or that the amyloid formation process is intracellular. In both alternatives it is likely that fibrils serve as nidus for further amyloid growth.

### SAA Measurements

**Figure 8:**
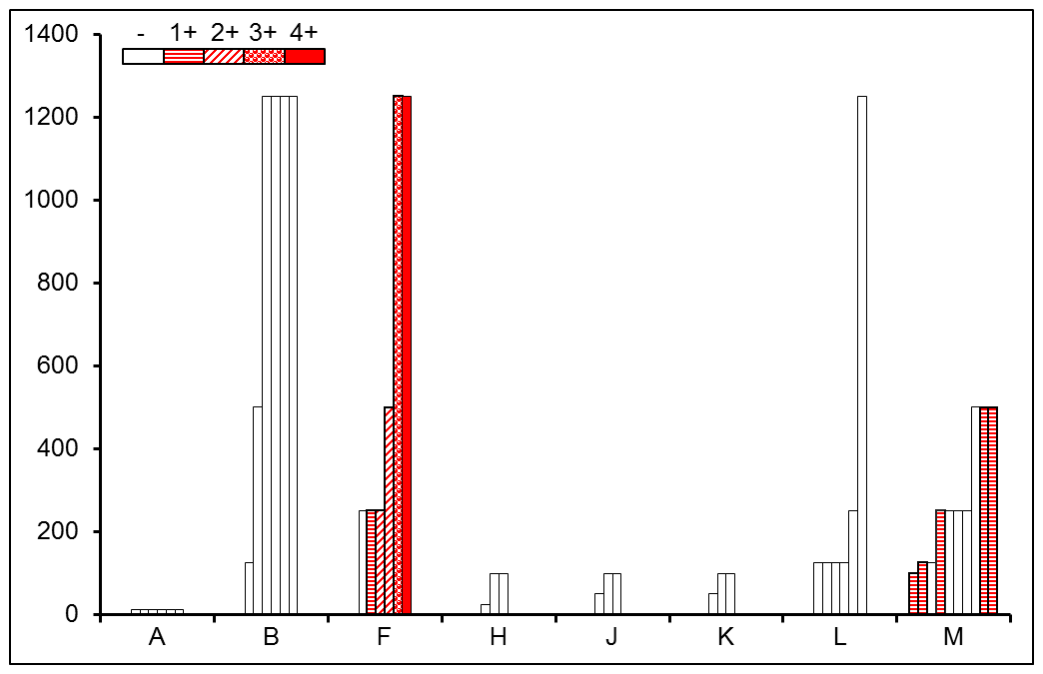
SAA concentrations were determined in serum. Group A untreated controls. Group B was injected with AgNO_3_ day 0 and sacrificed day 4, group F was injected with AEF and AgNO_3_ day 0 and sacrificed day 4, groups H and J were injected with CL day -2 and sacrificed day 0 and 4, respectively, group K was injected with CL day -2 and AEF day 0, and sacrificed day 4. Group L was injected with CL day -2 and AgNO_3_ day 0 and sacrificed day 4. Group M was injected with CL day -2 and AgNO_3_ and AEF day 0 and sacrificed day 4. Empty bars indicate mice without amyloid; mice with amyloid graded +1 − 4+ are showed in red with different textures.

SAA concentration in untreated mice was determined to12.5 µg/ml by ELISA (group A), after AgNO3 injection 125 to 1250 µg/ml (group B) and after AgNO3 and AEF injections 250 to 1250 µg/ml (group F). Injection of CL *per se* caused a slight increase in SAA concentration (25 to 125 in group H, J and K), and addition of AgNO_3_ (group L) or AgNO_3_ and AEF injections (group M) resulted in further increase (Figure 8). In mice injected with AgNO_3_ and AEF (group F), the amount of amyloid increased with increasing SAA concentrations, while in mice from group M amyloid could be detected at lower SAA concentrations (Figure 8). Elimination of F4/80 macrophages by CL results in low concentrations of TNF-alfa and IL-6, both important for SAA elevation in acute phase response. Occurrence of amyloid in macrophage-depleted mice with impaired acute phase response indicates that amyloid formation does not dependent on high SAA concentration.

### Amyloidogenic Effects of Liposomes

An unexpected finding was that administration of CL *per se* accelerated amyloid formation in all inflamed mice, these deposits were scored 2+, (group O, Table 3, Figure 5, 6 and 7), and in 1 out of 6 uninflamed mice injected with AEF two days after macrophage depletion (group N, Table 3). This is a remarkable finding because no amyloid was expected to occur in mice treated with three injections of AgNO3 and sacrificed day 16 as shown from group C (Table 1). In a study by Mambule et al. it was shown that non-sulphate containing polysaccharide (PEG) have the potential to act as AEF [40]. It is possible that CL in the same way as PEG accumulates in lysosomes, and delay SAA degradation and this may be sufficient for amyloid formation.

## Conclusions

Taken together, our results show that amyloid or amyloid formation is extremely toxic to MZMs and in the absence of these macrophages amyloid formation is delayed. The cytotoxic effect may arise after perforations or distortion of membrane structures [41], [42], but we could not determine whether progressive MZM reduction depends on an intra- or extracellular process. It is possible that AEF binds to specific receptors present on cell surface and functions, as nucleus for SAA that flows through the spleen but it is also possible that AEF, phagocytosed by MZMs remains intracellular and seeds amyloid at this location. A finding of large intracellular amyloid deposits in MZMs supports the intracellular mechanisms of amyloid formation and cytotoxicity.

In contrast to MZMs, MMZMs appear to be insensitive to amyloid. This may in part be due to their location in the inner region of the marginal zone, but even though they became surrounded by amyloid this lead only to activation without induction of any cytotoxicity. After macrophage eradication MMZMs re-established before MZMs and now they seemed to participate in amyloid formation. However, this mechanism must be different from that used by MZMs because MMZMs were not affected by amyloid. This finding is interpreted as that all amyloid is formed extracellularly or that MMZMs can secrete amyloid that formed intracellulary.

RPMs appear also insensitive to amyloid and it seems that they do not participate in amyloid formation.

More studies are needed to elucidate how MZMs and MMZM participate in amyloid formation.

## Materials and Methods

### Animals

Outbreed female NMRI (Naval Medical Research Institute) mice 6–9 weeks old were obtained from B&K Universal (Södertälje, Sweden). Mice were housed under conventional conditions in groups of 5, with free access to standard chow (CRM, Expanded, Witham, England) and water. Animal experiments were approved by the animal ethics committee, Linköping University, Sweden.

### Preparation of AEF

AEF was prepared from amyloid laden mouse livers by homogenization in 0.15 M sodium chloride with 0.05 M sodium citrate and centrifugation at 15 000×g for 30 minutes at 4°C. Homogenization of the pellet and centrifugation was repeated 10 times. This was followed by three homogenizations in distilled water, and the third supernatant was used as AEF [20].

### Preparation of Liposomes Containing Clodronate

Multilayered liposomes were prepared as described by Van Rooijen et al. [43]. Briefly, for preparation of 4 ml liposomes with clodronate, 8 mg cholesterol (Sigma-Aldrich, St. Louis, USA) was dissolved in 10 ml chloroform in a round bottom flask and 0.86 ml phosphatidylcholine (Sigma-Aldrich) stock solution (100 mg/ml chloroform) was added. The chloroform phase was removed by low vacuum rotation evaporation. The phospholipid film was dispersed in 10 ml of 0.6 M clodronate (Sigma-Aldrich) in distilled water by gently rotation for 15 minutes. The suspension was kept under N_2,_ at room temperature, for 2 hours, followed by sonication in water bath sonicator (Elma Transsonic, Singen, Germany) for 3 minutes and then kept under N_2_ for additional 2 hours. Liposomes were centrifuged at 10, 000 g for 15 minutes in a 70 Ti fixed angle rotor Beckman (Beckman Instrument Inc., Fullerton, USA). The pellet was washed 3 times in sterile PBS and centrifuged at 25 000 g for 30 minutes between washes (Beckman). The pellet was resuspended in 4 ml sterile PBS and stored under N_2_, at 4°C. Liposomes were counterstained with 3% eosin in water and the size was determined under a light microscope.

### Quantification of SAA

SAA concentration in untreated mice from group A (n = 6) and study groups B (n = 6), F (n = 6), H (n = 3), J (n = 3), K (n = 3), L (n = 6) and M (n = 10) was measured with a commercially available ELISA kit (Tridelta Development Ltd, Maynooth, Ireland) according to the manufacturer’s instructions. Serum from uninflamed mice (groups A, H, J and K) was diluted 1∶200 and serum from inflamed mice (groups B, F, L and M) was diluted 1∶2000 in diluent buffer. Measurements were performed in a Victor 1420 multi label counter (PerkinElmer, Waltham, USA).

### Detection and Quantification of Amyloid

Frozen spleen and liver sections (10 µm) were placed on slides, air-dried and fixed in formalin for 15 minutes. Sections were stained with alkaline Congo red [44], examined in cross-polarized light and amyloid load was graded by two investigators unaware of given treatment. Congo fluorescence was used to visualize amyloid in immunolabelled sections.

### Antibodies

Primary antibodies used for this work are monoclonal rat anti-mouse F4/80 (AbD Serotec, Kidlington, UK), monoclonal rat anti-mouse MOMA-1 (BMA Biomedicals, Augst, Switzerland), biotinylated monoclonal rat anti-mouse ER-TR9 (BMA Biomedicals), monoclonal rat anti-mouse MARCO (AbD Serotec), rabbit anti-human Ki67 (Dako, Glostrup, Denmark), and secondary antibodies Alexa 488 donkey anti-rat IgG, Alexa 610 goat anti-rabbit IgG and Streptavidine Alexa 488 conjugate (Invitrogen, Eugene, USA), (Table 5).

**Table 5:** Description of antibodies used for the study.

Antibody	Specificity	Fixation	Dilution	Detection
Rat antimouse MOMA-1	MMZM	Formalin	1∶200	Alexa488
Biotinylated-rat antimouse ER-TR9	MZM	Acetone Formalin	1∶200	Streptavidin Alexa488
Rat antimouse MARCO	MZM	Formalin	1∶400	Alexa488
Rat antimouse F4/80	RPM	Formalin	1∶200	Alexa488
Rabbit antihuman Ki67	Proliferation	Formalin	1∶200	Alexa610

### Detection RPMs, MMZMs and MZMs in Spleen

Frozen spleen sections (10 µm) were air-dried on SuperFrost Plus slides (Menzel Gläser, Braunschweig, Germany) and fixed for 20 minutes in formalin, at room temperature (RT), or acetone at −20°C (Table 5). Incubation with primary antibodies was performed in a humidified chamber overnight, at RT. To visualize amyloid, sections were incubated with Congo red solution B (80% ethanol saturated with NaCl and Congo red) for 1 minute. Sections were rinsed in water, mounted with glycerol/TBS (1∶1) containing DAPI nuclear stain (1∶500) (Invitrogen) and examined in a Zeiss LSM 700 confocal microscope (Carl Zeiss Inc., Stuttgart, Germany). Images were analyzed with Zen 2009 light edition (Carl Zeiss), and Photoshop Elements 5.0 (Adobe Systems Inc. San Jose, USA).

### Determination of RPM, MMZM and MZM Area

Spleen sections from 3–5 randomly selected mice from groups A-P were immunolabelled for F4/80, MOMA-1 or ER-TR9 to identify RPMs, MMZMs and MZMs, respectively, followed by staining with Congo red solution B. Images of red pulp for RPMs were taken at ×63 magnification and of marginal zones for MMZMs and MZMs were taken at ×20 magnification. The labeled area was analyzed with Image J 1.42q (National Institute of Health, Bethesda, USA). RPMs were assessed in 15 randomly selected areas (15600 µm^2^) in each mouse. MMZMs were assessed in at least three randomly selected marginal zones in each mouse. Presence of amyloid influenced MZM and these cells were assessed in at least five marginal zones with amyloid and without amyloid, when applicable. MMZM and MZM were measured as percentage of marginal zone. Mean value from each individual is used for statistical calculation.

### Quantification of MZMs

To determine number of MZMs sections from 3 randomly selected mice from group A, B, D, E and 3 randomly selected mice with amyloid grade 1+ from group F were labeled with ER-TR9 antibodies and DAPI nuclear stain. Ten images from each section were taken at ×40 magnification. The cellular density was determined by counting the number of nuclei in 2 randomly selected predefined areas (2000 um^2^) of marginal zones in each section. The result is presented as % increase of cells for each group.

### Statistical Analysis

Fisher’s exact test was used for comparison between groups http://lansrud.com/fisher.htm. Two-sample independent T-test was used for comparison of macrophage areas with the OpenEpi version 2.3. http://www.openepi.com/Menu/OpenEpiMenu.htm.
